# Characterization and application of electrospun alumina nanofibers

**DOI:** 10.1186/1556-276X-9-44

**Published:** 2014-01-27

**Authors:** Jeon-Hee Kim, Seung-Joon Yoo, Dong-Heui Kwak, Heung-Joe Jung, Tae-Young Kim, Kyung-Hee Park, Jae-Wook Lee

**Affiliations:** 1 Department of Chemical and Biochemical Engineering, Chosun University, Gwangju 501-759, South Korea; 2 Department of Environmental and Chemical Engineering, Seonam University, Namwon 590-711, South Korea; 3 Department of Life Science and Biotechnology, Shingyeong University, Hwaseong 445-741, South Korea; 4 Department of Environmental Engineering, Chonnam National University, Gwangju 500-757, South Korea; 5 Research Institute of Advanced Engineering Technology, Chosun University, Gwangju 501-759, South Korea

**Keywords:** Alumina nanofibers, Electrospinning, Adsorption, Pseudo-second-order kinetic

## Abstract

Alumina nanofibers were prepared by a technique that combined the sol–gel and electrospinning methods. The solution to be electrospun was prepared by mixing aluminum isopropoxide (AIP) in ethanol, which was then refluxed in the presence of an acid catalyst and polyvinylpyrolidone (PVP) in ethanol. The characterization results showed that alumina nanofibers with diameters in the range of 102 to 378 nm were successfully prepared. On the basis of the results of the XRD and FT-IR, the alumina nanofibers calcined at 1,100°C were identified as comprising the α-alumina phase, and a series of phase transitions such as boehmite → γ-alumina → α-alumina were observed from 500°C to 1,200°C. The pore size of the obtained γ-alumina nanofibers is approximately 8 nm, and it means that they are mesoporous materials. The kinetic study demonstrated that MO adsorption on alumina nanofibers can be seen that the pseudo-second-order kinetic model fits better than the pseudo-first-order kinetic model.

## Background

In recent years, ceramic with nanostructures has attracted a lot of attention and is being used in the fields of electronics, information technology, and communications [1]. It has found wide application in other areas as well, including the mechanical and chemical sciences and electrical, optical, and electrochemical energy sectors as effective electrode materials [2,3]. Among various chemical or physical synthetic methods, the electrospinning method is a popular one and involves the use of an electrically charged jet of polymer solution to form the nanofibers. The method can be described as follows. A high voltage is applied to the ceramic material solution with a polymer, and an electric field is generated between the tip of the syringe containing the solution and the collector. The solution is ejected in the form of a jet by electrical repulsion onto the collector, and fibers of nanoscaled diameters with inorganic precursor are formed [4]. The precursor nanofibers at high temperature are calcined to remove the polymers, and ceramic phase is obtained. This technique has been applied for the preparation of various metal oxide and ceramic nanofibers as well [5,6], which included TiO_2_[7], ZnO [8], SnO_2_[9], BaTiO_3_[10], and Al_2_O_3_[2-6,11].

Alumina (Al_2_O_3_) is one of the most important types of ceramic and is applied to the areas of catalysis, reinforcing components, electronic device fabrication, microelectronics, optics, and fire protection [12]. Most recently, alumina has been explored as effective electrode material for electrochemical energy storage device [13-15]. Al_2_O_3_ has specific physical, chemical, and mechanical properties, and during the process of forming the stable α-Al_2_O_3_, gibbsite is transformed to boehmite and then to a variety of metastable intermediate structures such as χ-, γ-, κ-, δ-, θ-alumina, depending on the temperature [16,17].

The main objective of the study is to investigate the calcination conditions on morphological appearance and crystal structure of the resulting alumina and the adsorption property of alumina calcined at different temperatures. Therefore, we investigated the synthesis of alumina nanofibers using a technique that combined the sol–gel and electrospinning methods using aluminum isopropoxide (AIP), an organometallic compound, as the precursor and polyvinylpyrolidone (PVP) polymer solution. The formation, morphology, and crystallinity of the electrospun alumina nanofibers were determined through thermogravimetric analysis (TGA), scanning electron microscopy (SEM), X-ray diffraction (XRD), and Fourier transform infrared (FT-IR) spectroscopy, Gas Chromatograph (Shimadzu GC-2010 Plus AF) and the alumina nanofiber samples synthesized were evaluated by nitrogen adsorption/desorption analysis. In addition, different phase alumina nanofibers were applied for the adsorption of methyl orange dye (MO) solution.

## Methods

The PVP (MW = 1,300,000; Kanto, Japan), AIP (C_9_H_21_O_3_Al) (>97.0%; Sigma-Aldrich Corporation, St. Louis, MO, USA), ethanol (94.0%; Daejung, Korea), and nitric acid (60%; Daejung, Korea) were obtained commercially and used as received without further purification. All the equipment used in the study was thoroughly cleaned prior to the experiments. A typical synthesis run was as follows: A certain amount of nitric acid and 10 mmol of the aluminum precursor AIP were added to 20 mL of ethanol, and the solution was stirred vigorously. The final composition of the mixed solution was such that the molar ratio of AIP/nitric acid/ethanol was 1:*m*:34, where *m* (=2.57) is the molar ratio of the acid (HNO_3_) to the alkoxide [17]. The mixture was covered with polyethylene (PE) film and then stirred vigorously at room temperature for at least 5 h. The PVP solution (10 wt.%) was prepared by dissolving the PVP polymer powder in ethanol under constant and vigorous stirring. The weight ratio of the polymer to the aluminum precursor was maintained at 3:1. The AIP and PVP solutions were then mixed, and the resulting AIP/PVP solution was loaded into a 10-mL syringe (SGE LL type) that was fitted with a metallic needle. The positive terminal of a variable high-voltage power supply was connected to the metallic needle and the negative terminal to a rotating collector (speed = 200 rpm) that was covered with the aluminum foil and served as the counter electrode. During a typical procedure, the voltage and the feeding rate were kept at 18 kV and 1.5 mL/h, respectively. The distance between the needle tip and the collector was maintained at 18 cm.

After the electrospinning was complete, the as-electrospun nanofibers were dried at 80°C for 24 h. Some of the dried nanofibers were used for the characterization by TGA, SEM, energy-dispersive X-ray spectroscopy (EDX), FT-IR spectroscopy, XRD, gas chromatography (Shimadzu GC-2010 Plus AF, Nakagyo-ku, Kyoto, Japan), and Brunauer-Emmett-Teller (BET) analysis. The remaining as-spun AIP/PVP composite nanofibers were calcined at different temperature (500°C to 1,200°C) for 2 h each at a heating rate of 5°C/min in order to obtain alumina nanofibers. Also, calcined alumina nanofibers were used for the characterization analysis and adsorption properties. As mentioned previously, the morphology of the fibers was examined by SEM (S4800, Hitachi Ltd., Tokyo, Japan). The diameters of the nanofiber were calculated from the SEM images using the Image J (National Institutes of Health, USA) software. The X-ray diffraction data was obtained with an X'Pert PRO MPD (PANalytical, B.V., Almelo, The Netherlands) diffractometer using Cu Kα radiation. FT-IR spectroscopy was performed on the samples using a NICOLET6700 (Thermo Scientific, Waltham, MA, USA) spectrometer that had a KBr beam splitter (operational wavelength range = 7,800 to 350 cm^−1^). TGA (STARSW, Mettler) was conducted up to 1,000°C with a heating rate of 5°C/min under a nitrogen and air atmosphere to evaluate the thermal behavior of the component nanofibers. The specific surface area and pore volume of the prepared alumina nanofibers were measured using the BET equation and the Horvath-Kawazoe (HK) method (ASAP2020, Micromeritics) after preheating the samples to 150°C for 2 h to eliminate adsorbed water. The pore size distributions were obtained by applying the HK method (micro-pore) to the nitrogen adsorption isotherms at 77 K using the software ASAP 2020.

## Results and discussion

**Figure 1:**
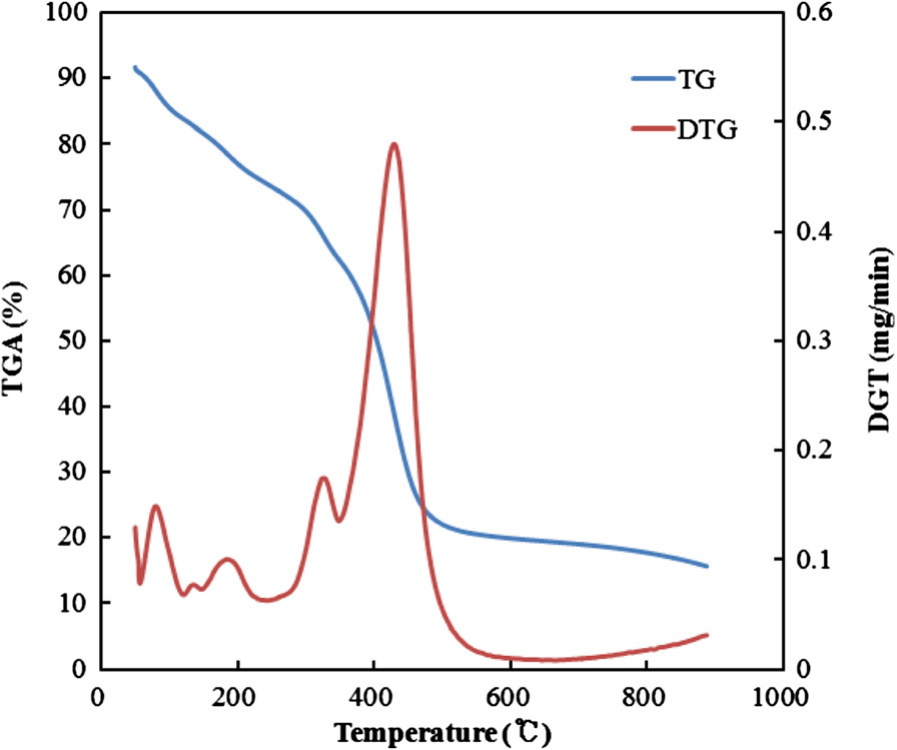
Thermogravimetric curve and derivative weight loss curve of the as-electrospun AIP/PVP composite nanofibers.

Figure 1 shows the results of the thermogravimetric curve and the derivative weight loss curve of the as-electrospun PVP and AIP/PVP composite nanofibers. At the AIP/PVP composite nanofiber curve, endothermic and exothermic peaks were observed with a corresponding weight loss of about 20%, in the region extending to 175°C. These peaks were attributed to the vaporization of physically absorbed water and the removal of any remaining solvent from the composite fibers. In the region extending from 200°C to 300°C, an endothermic and exothermic peak was observed that was associated with a weight loss of 30%. This observation was in accordance with the previous report by Kang et al. [18,19] that a weight loss resulted from the decomposition and burning of the PVP polymer fibers. The peaks were observed between 300°C and 400°C, and the weight loss associated with these peaks was 60% and indicated the complete combustion of the PVP polymer fibers and the organometallic compound of AIP. In contrast to a study on sol–gel process without PVP performed by Xu et al. [17], the prominent exothermic peak was observed at 429°C and indicating the complete combustion of the PVP polymer fibers.

**Figure 2:**
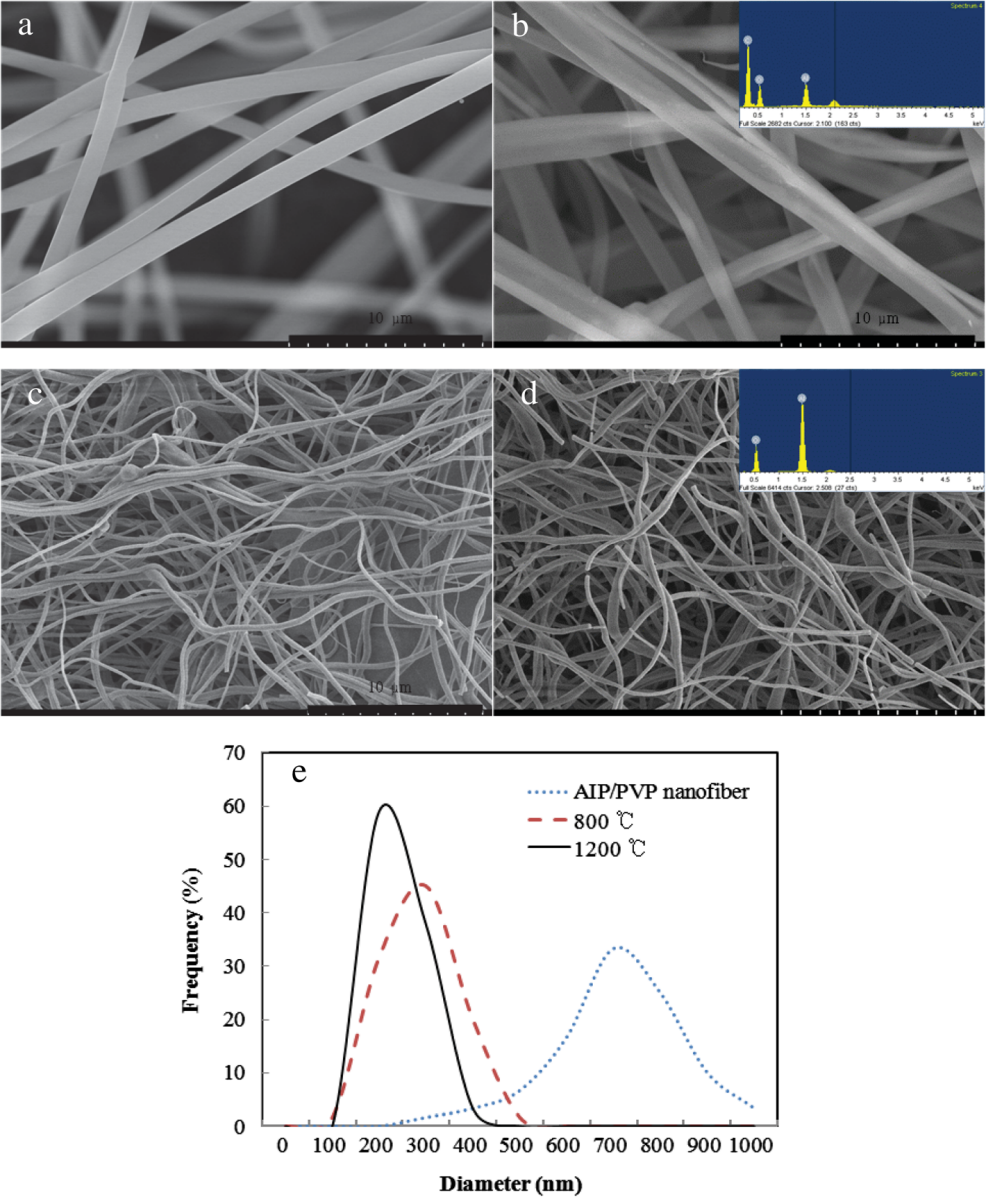
**SEM images and diameter distributions.** SEM images of as-electrospun PVP **(a)**, as-electrospun AIP/PVP nanofibers **(b)**, nanofibers calcined at 800°C **(c)** and 1,200°C **(d)**. Diameter distributions **(e)**. The inset shows EDX quantification.

The SEM micrographs of the composite nanofibers show that the as-electrospun fibers as well as those calcined at 800°C and 1,200°C had similar morphologies (Figure 2). As can be readily seen, in addition to their shapes, the continuous morphology of the as-electrospun composite nanofibers was maintained in the calcined nanofibers as well. Cylindrical nanofibers with diameters in the range of 276 to 962 nm could be successfully prepared using AIP as the precursor (Figure 2b). The diameter of these nanofibers decreased after calcinations at 800°C and 1,200°C, and alumina nanofibers with diameters of 114 to 390 nm (Figure 2c) and 102 to 378 nm (Figure 2d) were obtained after the respective heat treatments. In addition, as the calcination temperature increased, the average diameter of the alumina nanofibers decreased continuously, indicating that the organic groups further decrease in diameter for an increase in the calcination temperature beyond 1,200°C. The alumina nanofibers fabricated in this study were thinner and had narrower diameter distributions than those reported by Kang et al. [8]. From the EDX analysis, as-electrospun AIP/PVP nanofibers calcined at 800°C and 1,200°C showed C, O, and Al, and only Al and O, respectively. This result indicates the formation of pure alumina nanofibers. As-electrospun AIP/PVP nanofibers calcined at 800°C had 67.13% of C, 29.37% of O, and 3.5% of Al, and those calcined at 1,200°C had only 61.38% of O and 38.62% of Al, respectively.

**Figure 3:**
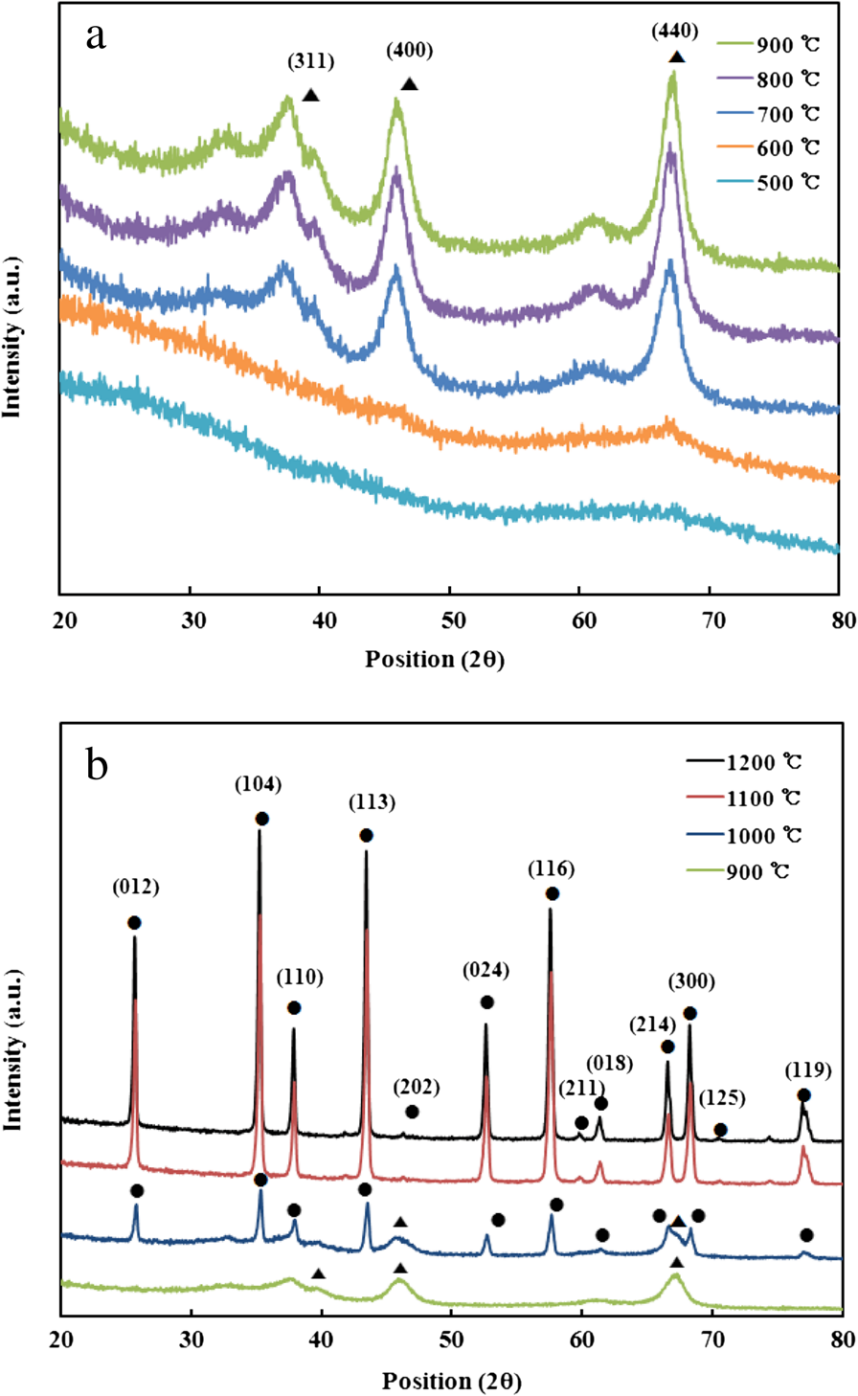
**XRD spectra of alumina nanofibers.** Calcined at 500°C, 600°C, 700°C, 800°C, and 900°C **(a)**, and 900°C, 1,000°C, 1,100°C, and 1,200°C **(b)**.

Figure 3 shows the XRD spectra of the alumina nanofibers calcined between 500°C and 1,200°C. There was also no distinct diffraction peak appearing for the samples calcined at 500°C and 600°C, and phase structure was found to be amorphous/microcrystalline. However, with the increase of calcination temperature up to 900°C, the typical peak of γ-Al_2_O_3_ was displayed with strong diffraction intensity. The γ-phase structure became weak when the temperature was above 1,000°C and completely disappeared at 1,100°C. The XRD spectrum of the sample calcined at 1,200°C indicated that α-alumina phase was formed. All the observed diffraction peaks matched well with those reported by Shanmugam et al. (JCPDS card no. 42-1468) [13]. From the above results, the phase transition of alumina nanofibers in this study can be shown as follows: amorphous/microcrystalline → γ-Al_2_O_3_ → α-Al_2_O_3_. In the process of heat treatment, the trihydroxide undergoes a series of transformation because of the water loss from hydration.

**Figure 4:**
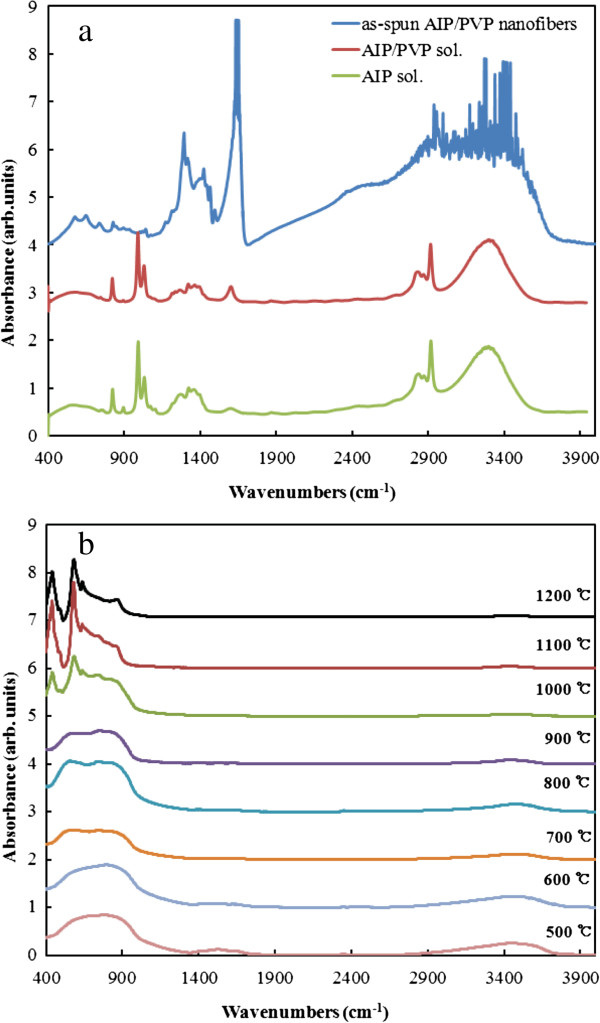
**FT-IR spectra of alumina fibers.** AIP solution, AIP/PVP solution, and as-electrospun AIP/PVP composite nanofibers **(a)**, and alumina nanofibers calcined at different temperatures **(b)**.

Figure 4 shows the FT-IR spectra of the alumina fibers obtained after calcination of the composite fibers at 500°C to 1,200°C, AIP solution, AIP/PVP solution, and as-electrospun composite fibers. Three characteristic peaks at 634, 581, and 440 cm^−1^ for alumina nanofibers calcined at 1,000°C, which it was confirmed α-phase alumina (Figure 4b), were observed, indicating Al-O bending and Al-O stretching. These peaks can be attributed to the presence of alumina; this conclusion is also supported by results of the XRD analysis [13].

**Figure 5:**
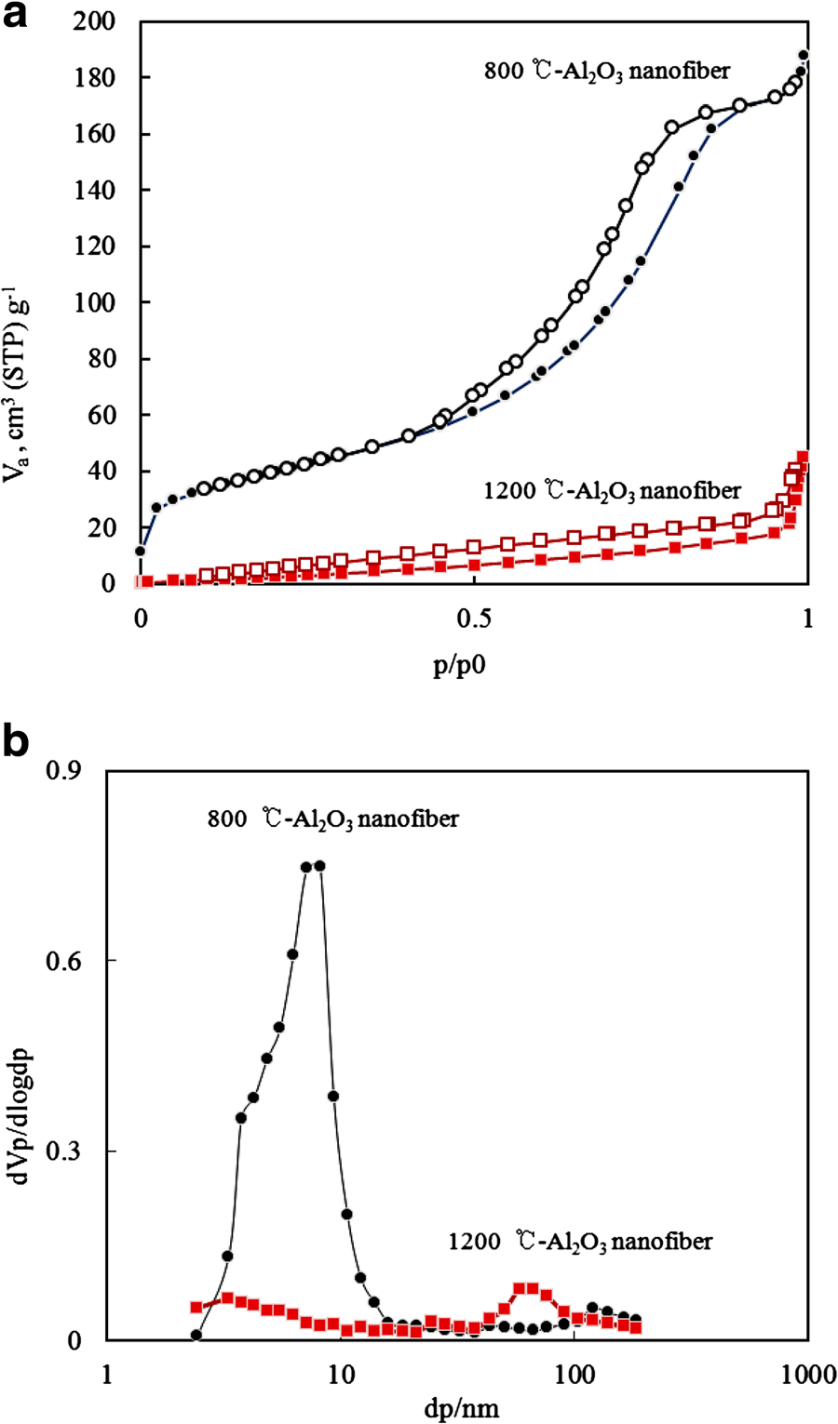
**N**_**2 **_**adsorption-desorption isotherms (a) and pore size distribution curves (b) of alumina nanofibers.** Calcined at 800°C and 1,200°C.

The nitrogen adsorption and desorption isotherms and the corresponding pore size distribution of the synthesized alumina nanofiber calcined at 800°C and 1,200°C temperatures are shown in Figure 5. As observed in Figure 5a, both the isotherms were types IV and V, which were related to the mesoporous structure. However, the types of hysteresis loops were different from each other as the calcination temperatures changed. The hysteresis loop type of the alumina nanofiber calcined at 800°C and 1,200°C were H2 and H4 [20]. The surface area of two samples calcined at 800°C and 1,200°C were 177.8 and 42.7 m^2^/g.

Simple adsorption kinetic experiments were performed at concentrations of 10 mmol/L for MO with α- and γ-alumina nanofibers. In each concentration, a series of 5 mL of MO solutions with 3 mg of alumina nanofiber were placed in residual MO concentrations, and *C*_t_ was determined at 460 nm. The pseudo-first-order kinetic model is described by the following equation [20]:

(1) $$\ln\;\left(q_{e}-q_{t}\right)=\ln q_{e}-\frac{k_{1}}{2.303}t,$$

where *q*_e_ and *q*_*t*_ are the capacity of metal ions adsorbed (millimole per gram) at equilibrium and time *t* (minute) and *k*_1_ is the pseudo-first-order rate constant (per minute). The pseudo-second-order model refers that the adsorption process is controlled by chemisorption through sharing of electron exchange between the solvent and the adsorbate [21]. The adsorption kinetic model is expressed as the following equation [20]:

(2) $$\frac{t}{q_{t}}=\frac{1}{k_{2}q_{e}^{2}}+\frac{1}{q_{e}}t.$$

**Figure 6:**
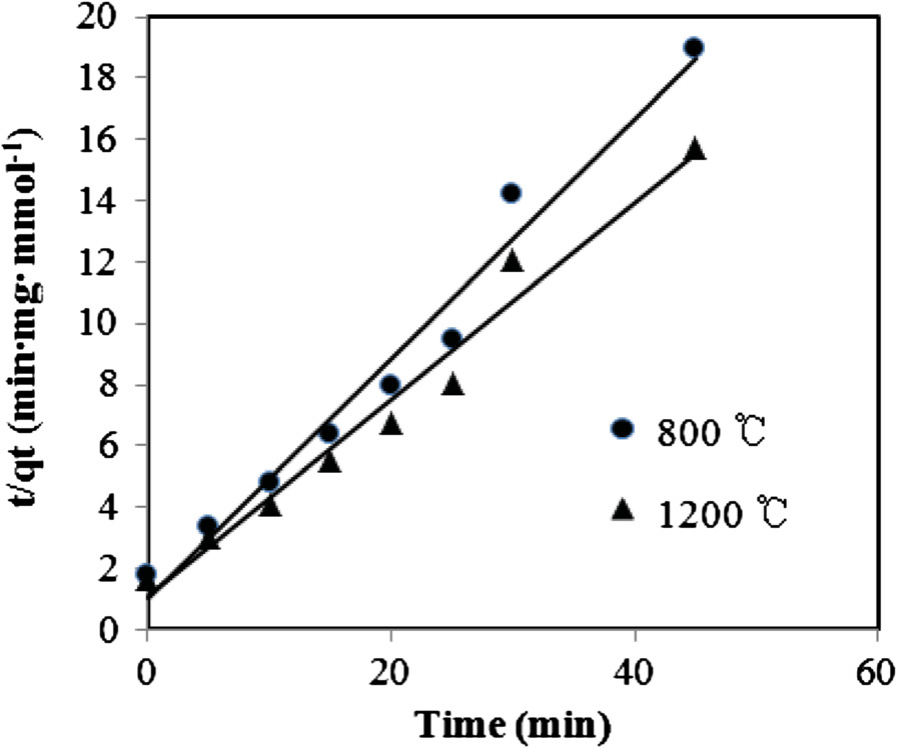
Pseudo-second-order adsorption kinetics of alumina nanofibers calcined at 800°C and 1,200°C.

The values of *k*_2_ and *q*_e_ can be calculated from the intercept and the slope of the linear relationship, Equation 2, between *t*/*q*_*t*_ and *t*. The curves of the plots of *t*/*q*_*t*_ versus *t* were given in Figure 6, and the calculated *q*_e_, *k*_1_, *k*_2_, and the corresponding linear regression correlation coefficient *R*^2^ values are summarized in Table 1. From the relative coefficient (*R*^2^), it can be seen that the pseudo-second-order kinetic model fits the adsorption of MO on alumina nanofibers better than the pseudo-first-order kinetic model.

**Table 1:** Kinetic parameters for the adsorption of MO on alumina nanofibers

**Calcination temperature (°C)**	**Pseudo-first-order kinetic model**			**Pseudo-second-order kinetic model**		
	***k***_**1 **_**(min**^**−1**^**)**	***q***_**e **_**(mol g**^**−1**^**)**	***R***^**2**^	***k***_**2 **_**(g mol**^**−1**^ **min**^**−1**^**)**	***q***_**e **_**(mol g**^**−1**^**)**	***R***^**2**^
800	0.208	1.560	0.7757	0.458	3.220	0.9999
1,200	0.048	1.818	0.6986	0.328	3.802	0.9995

## Conclusions

Alumina nanofibers were prepared by combining the sol–gel and electrospinning methods using AIP as an alumina precursor. The thus-produced alumina nanofibers were characterized by TGA, SEM, XRD, FT-IR spectroscopy, and nitrogen adsorption/desorption analysis. It was found from the SEM images of the various samples that the fiber-like shape and continuous morphology of the as-electrospun samples were preserved in the calcined samples. The diameters of the fabricated alumina nanofibers in this study were small and in the range of 102 to 378 nm with thinner and narrower diameter distributions. On the basis of the results of the XRD and FT-IR analysis, the alumina nanofibers calcined at 1,100°C were identified as comprising the α-alumina phase. In addition, a series of phase transitions such as boehmite → γ-alumina → α-alumina were observed from 500°C to 1,200°C. Adsorption kinetic data were analyzed by the first- and second-order kinetic equations. The adsorption property of MO of the α- and γ-alumina nanofibers was confirmed on the basis of the pseudo-second-order rate mechanism. Mesoporous alumina nanofibers prepared by electrospinning method can be successfully applied for the removal of dye pollutant from aqueous solutions.

## Competing interests

The authors declare that they have no competing interests.

## Authors’ contributions

J-HK, S-JY, D-HK, H-JJ, T-YK, and K-HP participated in the material preparation and data analysis. J-WL drafted the manuscript. All authors read and approved the final manuscript.
